# Evaluation of the Morpho-Physiological, Biochemical and Molecular Responses of Contrasting *Medicago truncatula* Lines under Water Deficit Stress

**DOI:** 10.3390/plants10102114

**Published:** 2021-10-06

**Authors:** Loua Haddoudi, Sabrine Hdira, Mohsen Hanana, Irene Romero, Imen Haddoudi, Asma Mahjoub, Hatem Ben Jouira, Naceur Djébali, Ndiko Ludidi, Maria Teresa Sanchez-Ballesta, Chedly Abdelly, Mounawer Badri

**Affiliations:** 1 Centre of Biotechnology of Borj Cedria, Laboratory of Extremophile Plants, B.P. 901, Hammam-Lif 2050, Tunisia; loua.haddoudi@outlook.fr (L.H.); hdirasabrina@gmail.com (S.H.); punto80@yahoo.com (M.H.); Asma_inrat@yahoo.fr (A.M.); hatembj@gmail.com (H.B.J.); abdelly.chedly@gmail.com (C.A.); 2 Faculty of Mathematical, Physical and Natural Sciences of Tunis, Campus Universitaire El-Manar, University of Tunis El Manar, Tunis 2092, Tunisia; 3 Laboratory of Biotechnology and Postharvest Quality, Institute of Food Science, Technology and Nutrition (ICTAN-CSIC), Jose Antonio Novais, 10, 28040 Madrid, Spain; irene.romero@ictan.csic.es (I.R.); mballesta@ictan.csic.es (M.T.S.-B.); 4 Department of Ecosystem Biology, University of South Bohemia, Branisovska 1760, 370 05 Ceske Budejovice, Czech Republic; ihaddoudi@jcu.cz; 5 Centre of Biotechnology of Borj Cedria, Laboratory of Bioactive Substances, B.P. 901, Hammam-Lif 2050, Tunisia; dnaceur2014@gmail.com; 6 Plant Biotechnology Research Group, Department of Biotechnology, University of the Western Cape, Robert Sobukwe Road, Bellville 7530, South Africa; nludidi@uwc.ac.za; 7 DSI-NRF Centre of Excellence in Food Security, University of the Western Cape, Robert Sobukwe Road, Bellville 7530, South Africa

**Keywords:** *Medicago truncatula*, water deficit stress, morpho-physiological characters, biochemical parameters, *DREB1B*

## Abstract

*Medicago truncatula* is a forage crop of choice for farmers, and it is a model species for molecular research. The growth and development and subsequent yields are limited by water availability mainly in arid and semi-arid regions. Our study aims to evaluate the morpho-physiological, biochemical and molecular responses to water deficit stress in four lines (TN6.18, JA17, TN1.11 and A10) of *M. truncatula*. The results showed that the treatment factor explained the majority of the variation for the measured traits. It appeared that the line A10 was the most sensitive and therefore adversely affected by water deficit stress, which reduced its growth and yield parameters, whereas the tolerant line TN6.18 exhibited the highest root biomass production, a significantly higher increase in its total protein and soluble sugar contents, and lower levels of lipid peroxidation with greater cell membrane integrity. The expression analysis of the *DREB1B* gene using RT-qPCR revealed a tissue-differential expression in the four lines under osmotic stress, with a higher induction rate in roots of TN6.18 and JA17 than in A10 roots, suggesting a key role for *DREB1B* in water deficit tolerance in *M. truncatula*.

## 1. Introduction

Plants are often subject to several environmental stresses which negatively influence their growth, development, and productivity. Drought is one of the most harmful abiotic stresses, particularly in the Mediterranean basin, where arid climate causes water scarcity and high evapotranspiration [1,2]. Severe droughts may directly reduce or eliminate yields, leading to a decline in crop productivity by up to 50–70%, which may affect 40% of the global population [3]. Moreover, according to the World Health Organization, drought stress affects the livelihood of approximately 55 million people worldwide annually, and it is estimated that around 700 million people are at risk of displacement by 2030 because of droughts [3]. Hence, better understanding the plants’ responses to environmental stresses is a key prerequisite for the improvement of their drought tolerance and yield under water deficit [4]. Drought causes an array of morphological, physiological, biochemical, and molecular changes in plants [5]. At the morphological level, it disturbs the appearance of plants by reducing the number of shoots, shoot length, leaf number, leaf area, and plant biomass, and also by changing their root length and biomass. At the physiological level, photosynthetic and transpiration rates tend to decrease as a result of stomatal closure under water deficit, which results in a low leaf relative water content (RWC). Moreover, they influence the photosynthesis by reducing leaf carbon dioxide assimilation, chlorophyll and carotenoid contents, and accessory pigments [1,5].

At the biochemical level, drought stress induces a high level of lipid peroxidation, lipoxygenase activity, aldehyde, proline, soluble sugar contents, and electrolyte leakage [6,7,8]. At the molecular level, several regulatory gene products, such as CDPKs, MAPKs, HD-zip/bZIP, AP2/ERF, NAC, MYB, and WRKY, can cause changes in plants’ morphology or physiology by regulating signal transduction pathways or acting as transcription factors to regulate the expression of downstream genes and further enable plants to successfully survive under drought stress conditions [5,7]. To cope with the adverse effects of drought stress, plants trigger different adaptations, such as morphological and structural changes, the expression of drought-resistance genes, and the synthesis of phytohormones and osmotic regulatory substances to maintain growth and productivity under drought stress [1,5]. These adaptive strategies have evolved into three main survival mechanisms: stress avoidance, escape, and tolerance [9]. Drought avoidance occurs when plants succeed in maintaining a satisfactory water status by increasing their water use efficiency [10]; and this is also achieved by specialized adaptations in the plant’s architecture, such as the development or the reduction of specialized leaf surfaces to decrease the rate of transpiration and the increase in root length or density to use available water more efficiently [4]. Drought escape is a classical adaptive mechanism which involves rapid plant development to enable the completion of the full life-cycle prior to a coming drought event [11]. Drought stress tolerance is a result of the coordination of physiological and biochemical changes at the cellular and molecular levels: drought-tolerant plants are able to compensate decreased turgor using osmotic adjustment, or the production of metabolites that can help them repair drought-induced damage [12,13], or protein stabilization [10] and by accumulation of an abundance of late embryogenesis proteins coupled with an efficient antioxidant system [4]. Besides, the osmotic adjustment provided by the synthesis of osmoprotectants like proline, betaine, polyols, and soluble sugars may confer tolerance to drought by reducing the tissue osmotic potential and maintaining the water absorption from the external environment to maintain the cellular turgidity [5,12,14]. The accumulation of osmoprotectants in plants is strongly correlated with the resistance or tolerance of plants to abiotic stresses under restricted water availability [15,16], since it contributes to adjusting the cellular osmotic potential, reducing the toxicity of reactive oxygen species, maintaining membrane integrity, and enzyme/protein stabilization [1]. Hence, the identification of these compounds provides a promising connecting link that could bridge the gap between genotype and morpho-physiological traits. As osmotic regulating substances, soluble sugars and soluble protein content are considered as important indicators of the physiological status of plants related to drought tolerance [17,18]. Genes regulating the levels of osmoprotectants are highly stress-responsive and were among the first stress-inducible transcripts reported in the literature [19]. In particular, the expression of these genes causes physiological and biochemical changes, i.e., an increase in sugar and soluble proteins contents, and changes in the composition of lipid membranes and the proline level [20,21]. Some of the regulatory genes of drought stress responses are transcription factors [22] that can be strongly induced by water deficit stress and whose expression can regulate the expression of various genes associated with drought responses [5].

Previous studies of the expression of *DREB1B* genes were characterized in *Arabidopsis*, soybean, rice, and other plant species [23,24], exploring the potential use of *DREB1B* as candidate genes in responses to numerous stresses, mainly to salt and drought stress, although there is evidence for the regulation of their expression by drought in the roots of some plant species. Furthermore, a sequence analysis of these genes demonstrated that the candidate gene is a single-exon gene, while its duplication has generated a small multigene family during the evolution of species [25].

Moreover, DREB proteins were shown to activate the expression of genes involved in osmoprotectant biosynthesis pathways, such as the LEA proteins or soluble sugars, which are related to improved salt and drought tolerance [26]. These genes regulate osmoprotection, water and ion movements, a variety of functional and structural stress-induced proteins, signal perception and transduction, free radical scavenging, and many other components [27].

Most legumes (Fabaceae) are sensitive to drought stress. *Medicago truncatula* L. is an important forage leguminous plant of Mediterranean origin. It has several economic and ecological characteristics that made it a model forage crop in many farming systems and breeding programs for legume crops. Such characteristics include its small diploid sequenced genome (−500 Mb) [28], its high efficiency of genetic transformation, a wide biological diversity, and its high conservation synteny with cultivated legumes such as *Lotus japonicus*, alfalfa, soybean, and common bean [29]. Additionally, the use of tolerant genotypes is one of the strategies to deal with water shortage in the agricultural sector. Selecting drought-tolerant cultivars by examining their performance under water deficit stress conditions will be useful in sustaining agricultural productivity under water limitations [30]. Since plant performance is influenced by physiological and biochemical traits, these traits can be used as a tool to identify, screen, and select drought-tolerant plants.

In this context, the objective of the present study was to compare the effect of drought stress on plant growth and development, the antioxidant activity, and the osmoprotectant contents in the four lines of *M. truncatula*, aimed at selecting tolerant lines that can grow and yield satisfactorily in drought-prone areas. Moreover, an expression profiling of *MtDREB1B* in response to osmotic stress was carried out to provide insights into the molecular basis of drought tolerance mechanisms mediated by this gene in legumes.

## 2. Results

### 2.1. Effects of Water Deficit Stress on Morpho-Physiological Traits

The results from the ANOVA showed a significant difference between lines, water treatments, and the interaction of lines × water treatments. The water deficit treatment attributed most of the variation in measured traits (Table 1).

The variation of 13 traits among those measured was dependent on the treatment effect (Table 1). Furthermore, out of the 13 traits, 8 are significantly influenced by the interaction of line × treatment.

#### 2.1.1. Measurement of Plant Growth Parameters

Most of the measured traits showed significant differences between lines JA17, TN6.18, A10, and TN1.11 in both control treatment and 30% of field capacity.

The results showed that the water deficit significantly reduced the length of stem for the four lines of *M. truncatula*. The JA17 line showed the lowest reduction (38.01%) of length of stem, followed by TN6.18 and A10 (43.75% and 48.14%, respectively), and a height mean reduction was recorded for TN1.11 (Table 2).

**Figure 1:**
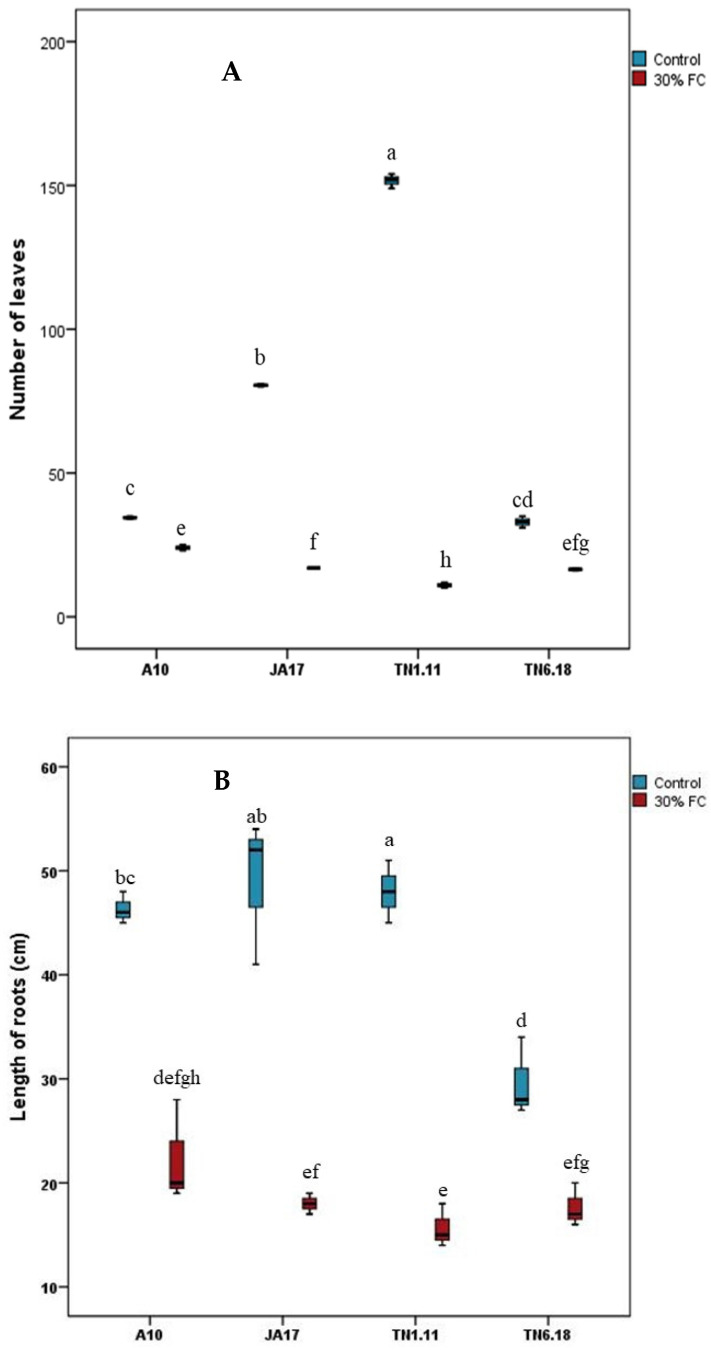
Number of leaves (**A**) and length of roots (**B**) for the four lines of *M. truncatula* under control treatment and 30% FC. Means followed by the same or a common letter (s) are not significantly different among the studied lines for each trait according to Duncan’s test at 5%.

Furthermore, under water deficit stress, the lowest decrease in the number of leaves were recorded for the TN6.18 and A10 lines, while the strongest reductions were registered for the JA17 and TN1.11 lines (Figure 1). Similarly, the JA17 and TN1.11 lines showed the most pronounced reductions for length of roots, at 66.67% and 67.37%, respectively, while the lowest value was registered for TN6.18 (39.51%). The results suggest that TN6.18 is more water-use efficient than the other studied lines (Figure 1).

#### 2.1.2. Biomass Production

Plant growth parameters play an important role as a selection criterion to assess the tolerance to drought stress in crop plants.

#### 2.1.3. Fresh Biomass

**Figure 2:**
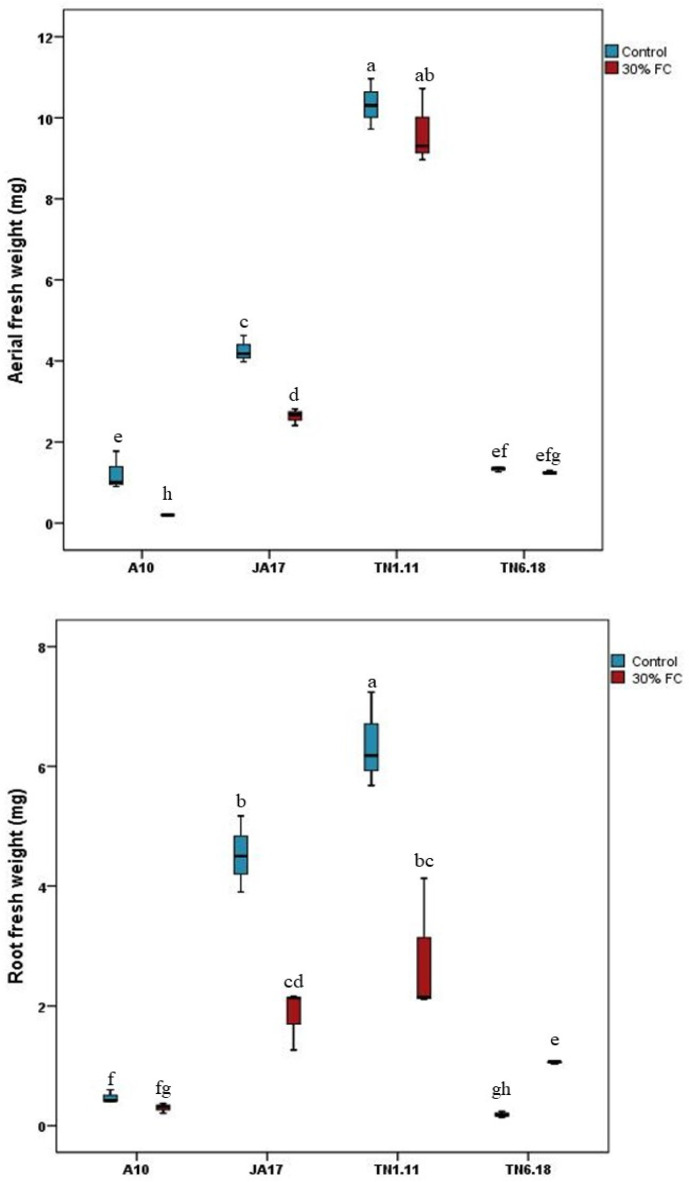
Means of shoot and root fresh weights for the four lines of *M. truncatula* under control and 30% FC. Means followed by the same or a common letter (s) are not significantly different among the studied lines for each trait according to Duncan’s test at 5%.

Under 30% FC, JA17, A10, and TN1.11 showed a decrease of 38.21, 41.61, and 6.45% in their shoot fresh weight, respectively, with the biggest effect observed for A10 compared to the control (Figure 2). Furthermore, the water deficit stress induced a reduction in root fresh weight, with 59.07%, 36.07%, and 56.20% for JA17, A10, and TN1.11, respectively.

Nevertheless, in comparison to controls, water deficit had the least impact on the aerial fresh weight (3.17% of reduction) of TN6.18, with an increase in root fresh weight (20.12%). These results demonstrate the physiological differences between the genotypes, in relation to root growth as a response to water stress. Thus, the TN6.18 line seems to be the most tolerant to water deficit stress compared to the other lines (Figure 2).

#### 2.1.4. Dry Biomass

**Figure 3:**
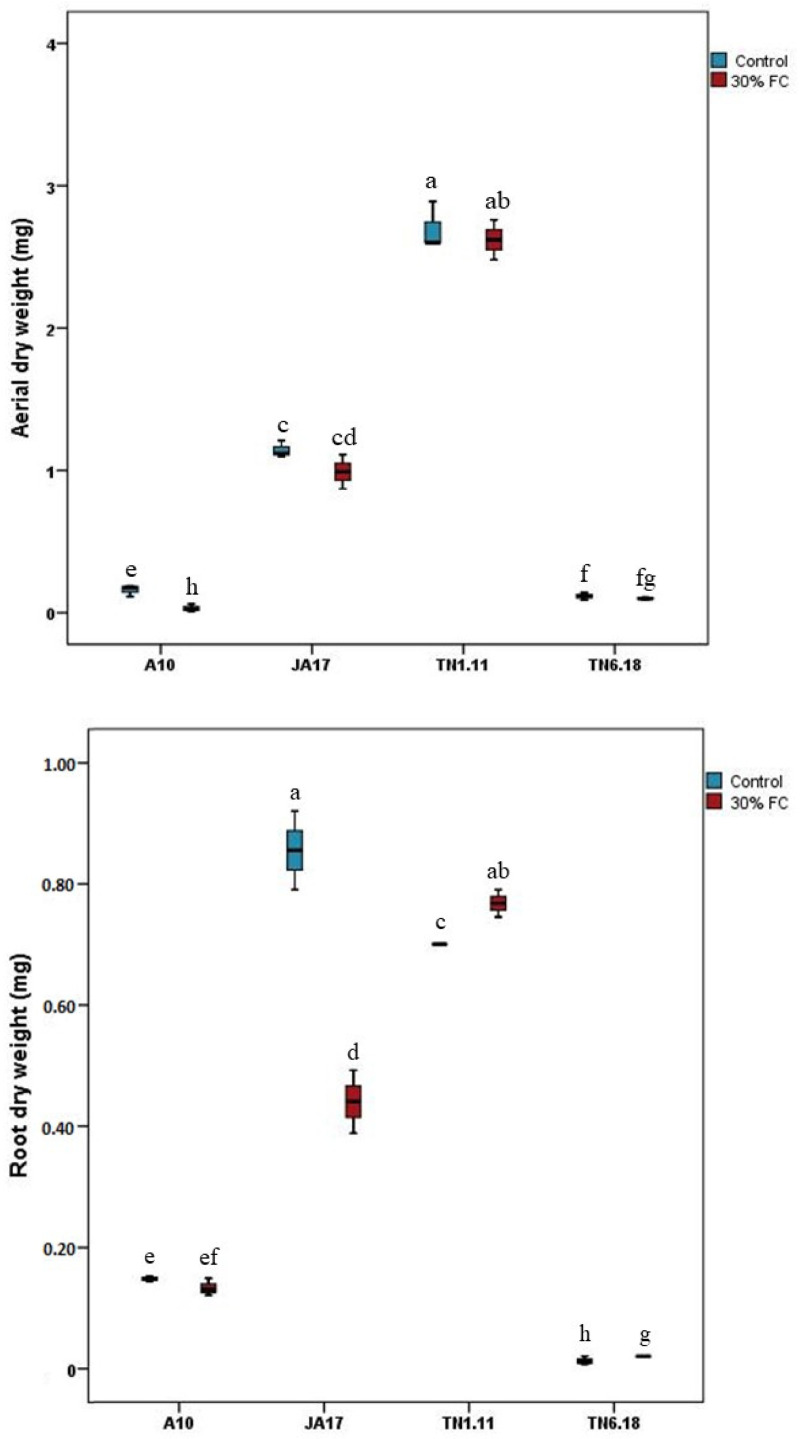
Means of dry weights (aerial and root) for the four lines of *M. truncatula* under control treatment and water deficit stress. Means followed by the same or a common letter (s) are not significantly different among studied lines for each trait according to Duncan’s test at 5%.

The 30% FC treatment induced reductions of 81.25, 16.66, 10.63, and 2.96% in A10, TN6.18, JA17, and TN1.11 shoot dry weights, respectively. These results represent a large reduction in the sensitive line A10 under water deficit stress, compared to the control plants (Figure 3). In contrast, TN1.11, JA17, and A10 showed a decrease of 81.42, 48.83, and 13.33%, respectively, in root dry weights, whereas TN6.18 exhibited an increase of 10.21% for root dry weight, suggesting that this line is more water-use efficient.

#### 2.1.5. The Root Dry Weight and Aerial Dry Weight Ratio (RDW/ADW)

**Figure 4:**
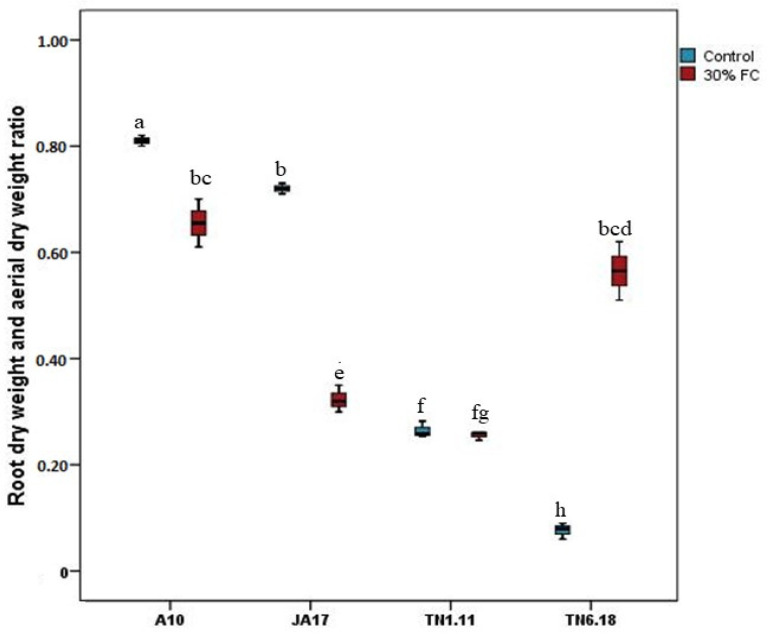
Means of root dry weight and aerial dry weight ratio for the four lines of *M. truncatula* under control treatment and water deficit stress. Means followed by the same or a common letter (s) are not significantly different among the studied lines for each trait according to Duncan’s test at 5%.

The root dry weight and aerial dry weight ratio ranged from 0.06 under control treatment to 0.09 under water deficit stress (Figure 4, Table S1). Under water deficit stress, TN6.18 showed an increase of this ratio.

#### 2.1.6. Heritability (*H*^2^) and Correlations between Traits

Broad sense heritability (*H*^2^) values of the measured traits ranged from 0.56 to 0.99 and from 0 to 0.98 under the control treatment and 30% FC, respectively (Table 3). Under the control treatment, high values of heritability (*H*^2^ > 0.4) were found for all measured characters. Moreover, high *H*^2^ were noted for the length of stems, number of leaves, aerial fresh weight, aerial dry weight, root fresh weight, root dry weight, and ratio under drought stress. Low levels (*H*^2^ < 0.2) were recorded for the number of axes and the length of roots traits.

Among the 81 possible correlations between the measured parameters, 19 correlations were significant under control treatment and 12 correlations were significant under 30% FC (Table 4). Among these correlations, 19 are positive under control conditions and 7 are positive under 30% FC. Positive correlations were noted between the fresh weights (AFW and RFW) and the number of leaves (NL) under both treatments.

The comparison between two correlation matrices of measured traits under both treatments (Table 3) showed that various correlations are specific in control treatment, such as the positive correlation between the length of roots (LR) and the root fresh weight (RFW), and specific correlations to water deficit have been noted between the number of leaves (NL) and the root dry weight and aerial dry weight ratio (RDW/ADW).

#### 2.1.7. PCA and Clustering Analysis Based on the DSI Values

**Figure 5:**
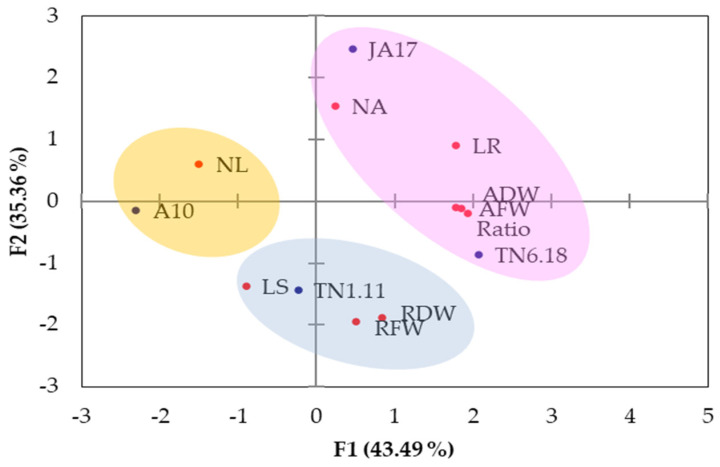
Distribution of the studied lines in the plan (1–2) of the principal components analysis based on drought sensitivity indices (DSI). Number of axes (NA), length of stems (LS, cm), number of leaves (NL), aerial fresh weight (AFW), aerial dry weight (ADW), length of roots (LR), root fresh weight (RFW), root dry weight (RDW), and aerial dry weight (RDW/ADW) ratio were assessed.

The first three principal components with eigenvalues > 1 explain the total variation (100%) among the studied lines. The first two axes of the PCA explain 43.49% and 35.36%, respectively, or 78.85% of the total variation. The first axis is positively correlated with the aerial fresh weight (AFW), aerial dry weight (ADW), length of roots (LR), and ratio, while it is negatively correlated with the length of stems (LS) and the number of leaves (NL) (Figure 5). The second axis is positively correlated with the number of axes (NA), while it is negatively correlated with the root fresh weight (RFW) and root dry weight (RDW).

**Figure 6:**
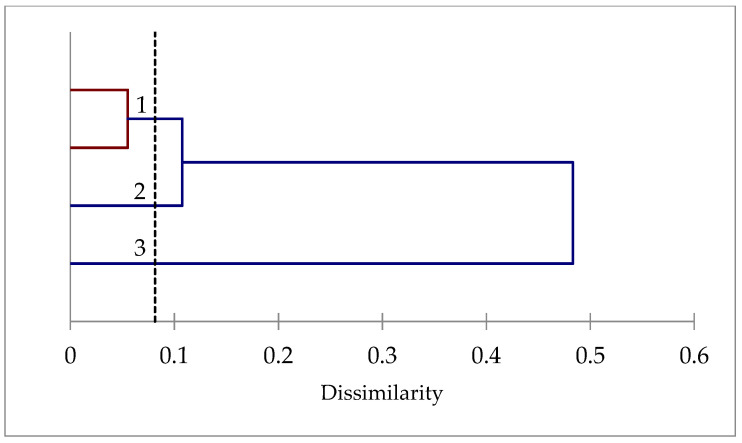
Dendrogram of *M. truncatula* lines based on Euclidean distances of the dissimilarity matrix using Ward’s method.

The results from the PCA showed that the studied lines formed three groups (Figure 6). A first group (the highest PC1 and PC2) is formed by the two tolerant lines TN6.18 and JA17, a second group (the intermediate PC1) contained TN1.11 with intermediate behavior, and a third group (the lowest PC1) is composed by the sensitive line A10.

### 2.2. Biochemical Analyses

Water deficit stress influenced malondialdehyde (MDA), while the protein content was influenced by the effects of line and the interaction of line × treatment. Furthermore, water treatments and the interaction of line × treatment influences soluble sugars content (Table 5).

#### 2.2.1. Lipid Peroxidation Assay

**Figure 7:**
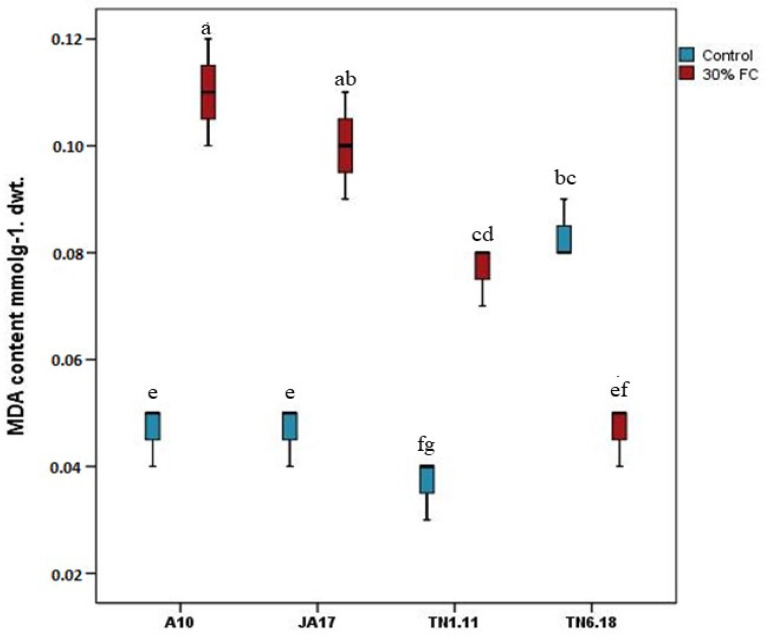
MDA content in *M. truncatula* studied lines under control treatment and 30% FC. Means followed by the same or a common letter (s) are not significantly different among the studied lines for each trait according to Duncan’s test at 5%.

The levels of lipid peroxidation, expressed as MDA content, were measured by estimating the MDA content in leaf tissues. Under control conditions, the highest accumulation of MDA was found for the TN6.18 (0.08 mmol/g DW), while the lowest value was registered for TN1.11 line (0.04 mmol/g DW) (Figure 7).

Our results showed that the concentrations of MDA in the three lines TN1.11, A10, and JA17 increased as a result of water deficit stress. However, TN6.18 exhibits a significant decrease of 38% in the MDA content under water deficit compared to the adequate water availability, reaching a level equal to that of the other lines under control conditions.

#### 2.2.2. Total Protein Content

**Figure 8:**
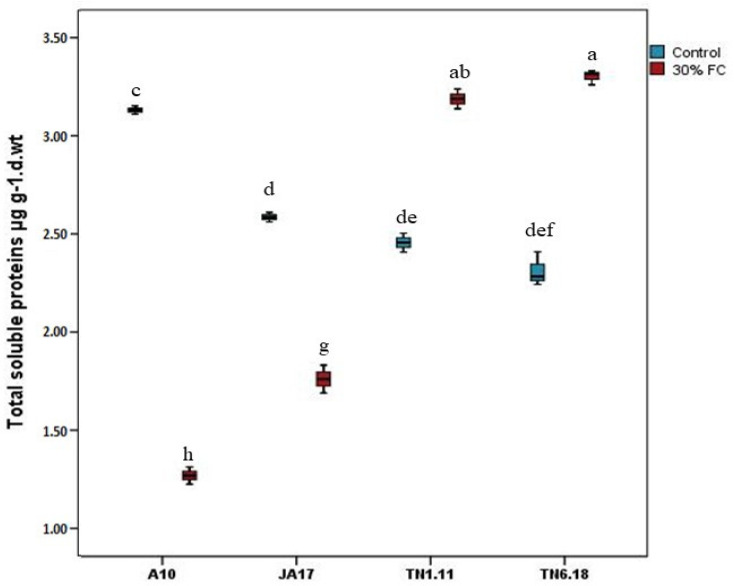
Variation of total protein content in *M. truncatula* studied lines under control treatment and 30% FC. Means followed by the same or a common letter (s) are not significantly different among the studied lines for each trait according to Duncan’s test at 5%.

Under adequate water availability, A10 had the highest value of protein content while the lowest value was observed for the JA17 line (Figure 8). The total protein content of the studied lines decreased by 57.22% in the A10 line, whereas no significant decrease was observed for JA17 under water deficit stress. The lines TN6.18 and TN1.11 had a significant increase in their total protein contents (by 43.44 and 39.30%, respectively) under water deficit, and its accumulation is considered an index of stress tolerance. Overall, although the TN6.18 line recorded a higher increase than the TN1.11 line, the lines JA17 and A10 exhibited the lowest values of protein content.

#### 2.2.3. Soluble Sugar Content

**Figure 9:**
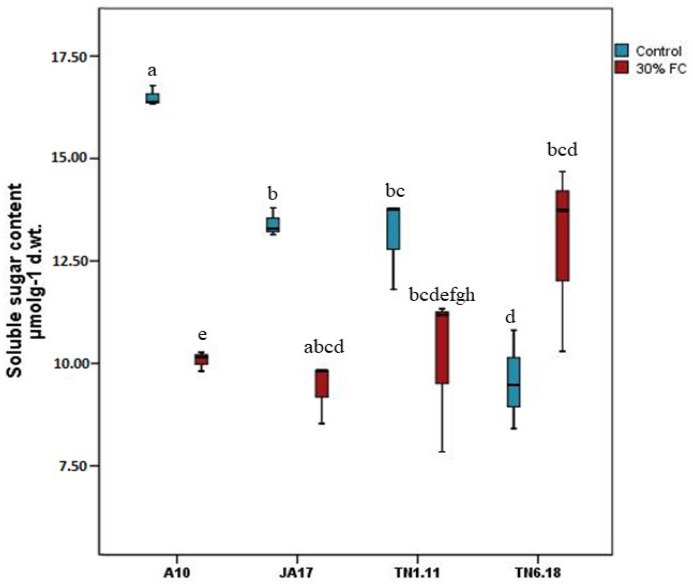
Variation in soluble sugar content for the studied lines of *M. truncatula* under control treatment and 30% of field capacity. Means followed by the same or a common letter (s) are not significantly different among the studied lines for each trait according to Duncan’s test at 5%.

In the control treatment, the A10 line exhibited the most abundant soluble sugars, followed by the JA17 and TN1.11 lines (Figure 9). Under drought stress, the content of total soluble sugars was reduced by 38.9 and 29.95% in A10 and JA17, respectively, while no significant decrease was noted for TN1.11. However, no significant increase in soluble sugar content was recorded in the TN6.18 line under water deficit (Figure 9).

### 2.3. DREB1B Expression Study under Osmotic Stress

**Figure 10:**
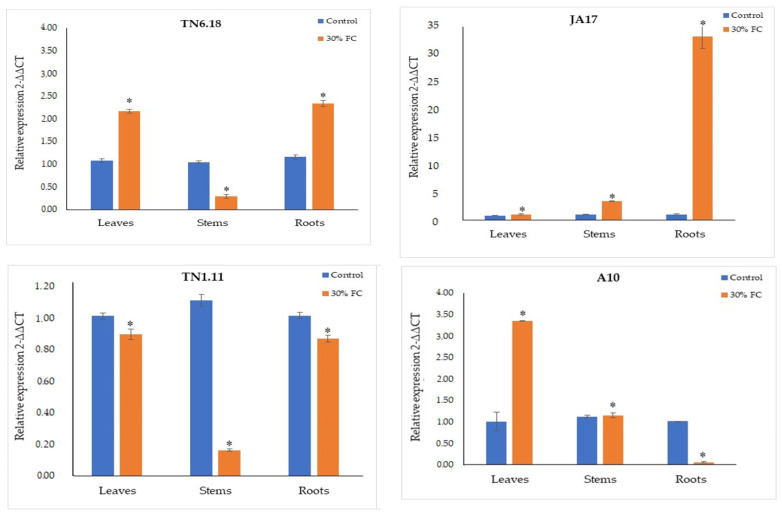
Variability of the expression analyses of *DREB1B* in leaves, stems, and roots of the studied lines of *M. truncatula* under control treatment and 30% FC. Asterisks indicate significant differences between treatments as estimated by ANOVA (* *p* ≤ 0.05).

The findings of the present study indicate that the expression patterns of *DREB1B* varied depending on the line and tissue factors (Figure 10 and Figure S1). The highest relative expression values were observed in the roots for tolerant lines under water deficit.

Indeed, the lines JA17 and TN6.18 showed a significant increase of expression levels, which were approximately 33- and 3-fold higher than the control plants, respectively, under water deficit stress, while a significant decrease of expression was observed for the A10 line (Figure 10).

The same pattern of expression was also observed for JA17 and TN6.18 under 100 mM NaCl, showing a significant increase of the expression value, of 16.40- and 3.64-fold, respectively. Nevertheless, a significant decrease of *DREB1B* expression was registered in roots for TN1.11 and A10, of 0.51 and 0.02-fold, respectively (Figure S1).

The ANOVA results reporting that the variation of the *DREB1B* gene’s expression showed a significant difference between lines, tissue, treatment, the interaction of line × tissue, the interaction of line × treatment, the interaction of tissue × treatment, and the interaction of line × tissue × treatment.

The line and tissue factors significantly explained most of the variation in the *DREB1B* gene’s expression for the studied lines (Table 6).

## 3. Discussion

Water deficit stress is a serious factor that negatively affects plant growth and yield in arid and semi-arid regions. In response to water deficit stress, plants activate their own drought response mechanisms, such as physiological and structural changes, the expression of drought-resistant genes, and the synthesis of hormones and osmotic regulatory substances to alleviate the water deficit stress [31]. The use of drought tolerant species is one of the strategies to deal with water shortage in the agricultural sector [2,3]. Since plant performance is influenced by morpho-physiological, biochemical, and molecular traits, these traits can be used as a tool to identify and select drought-tolerant plants. Therefore, in this study, we tried to use different indicators to select stress-tolerant genotypes in order to identify more drought-tolerant lines of *M. truncatula.*

### 3.1. Water Deficit Effects on the Growth and Biomass Production

Our results showed that the variation of measured traits was mainly influenced by the water deficit stress, which has caused a significant reduction in the leaf number, plant height, number of axes, and length of roots in all lines, with the highest reductions noted for the TN10 line. Accordingly, Hdira et al. [32] reported significant reductions for growth parameters in the same lines under salt stress conditions. The fresh and dry biomasses are generally considered as indicators to select tolerant lines at the seedling stage [33,34]. In this study, water deficit significantly decreased the shoot biomass in the sensitive line A10 compared with control plants, due to the lower turgor pressure caused by the low-soil water availability, which involves processes such as cell division and elongation [35,36].

In contrast to the sensitive TN10 line, an increase in root biomass under water deficit stress was observed in the tolerant line TN6.18 compared with control plants. This biomass distribution pattern revealed a different physiological behavior in the TN6.18 genotype, which increased the biomass allocation to the roots and reduced the biomass allocation to the leaves, which is a strategy to efficiently adapt to scarce precipitation in Mediterranean semi-arid environments [37,38]. These findings are consistent with Kim et al. [39] who reported that root density in tolerant rice genotypes increases in response to water deficit stress. At a reduced water potential, osmotic adjustment in the root system helps in maintaining some level of turgidity, and the water potential gradient is re-established for water uptake. These adjustments are responsible for the growth of roots under low water potential [40].

The reduction of available water in the soil induces osmotic stress and mediates the loss of cell turgor due to the lower water availability for cell expansion, thus decreasing the relative water content (RWC) [3], which is an important determinant of the metabolic activity and survival of leaf. RWC is used as an indicator of the water status of plants for drought tolerance [41,42]. Under water deficit stress (30% FC), there was a decrease of the RWC in all lines, while TN6.18 (with the least decline) exhibited a significantly better capacity to preserve water in its leaf tissues. These results are consistent with the findings of Ozkur et al. [43] and Hussain et al. [44], who reported that a lower reduction in RWC in *Capparis ovata* and maize, respectively, under water deficit is indicative of stress tolerance.

In the current study, most of the correlations between traits were positive, suggesting that most of the measured characters were similarly affected by water deficit stress. The dry and fresh plant weights were positively correlated with the numbers of leaves, suggesting that the reduction in biomass is related to the number of leaves for A10, as reported by Yu et al. [45] and by Hdira et al. [32], for Rosaceae species and *M. truncatula*, respectively. Among these correlations, the number of axes was negatively correlated with all the traits.

Heritability values that were higher to moderate were found for measured traits, indicating that much of their variation is under genetic control. High heritability values were found for the length of stems, number of leaves, aerial fresh weight, aerial dry weight, root fresh weight, and root dry weight under control treatment and water deficit stress. Consequently, these morpho-physiological traits can be considered as reliable descriptors for *M. truncatula* water deficit stress tolerance. These results are consistent with Arraouadi et al. [46], who reported that the length of stems, aerial dry weight, and root dry weight are better descriptors of salinity tolerance in Tunisian and reference lines of *M. truncatula.*

### 3.2. Biochemical Characterization

Plants accumulate compatible osmolytes as osmoprotectants to protect plants from water deficit stress [47]. The tolerant line TN6.18 responds to elevated water deficit stress by increasing the accumulation of soluble proteins and sugar contents in leaves, while a decrease in their contents was noted in JA17, TN1.11, and A10, suggesting that the highest drought tolerance for line TN6.18 might be due to its ability for osmotic adjustment. This increase in osmolyte accumulation contributes to the recovery of damaged proteins and protects the cells against oxidative damage by limiting the accumulation of ROS [48]. Furthermore, these compounds stabilize the osmotic balance of cells and help plants to withstand severe osmotic stress during their growth and development.

Our results are consistent with Zhang and Shi [49], who found that the tolerant line Longzhong of alfalfa exhibited higher levels of soluble protein and soluble sugar contents when exposed to water deficit stress. This increase has been described as a common behavior in drought-stressed plants due to their role in osmoregulation, amongst others [4]. A recent study of Echeverria et al. [50] focused on soluble sugars in relation to their role in the protection against water deficit stress. Free proline is well-known to increase in response to water deficit in plants, and recent evidence from metabolite measurements shows that water deficit leads to the elevation of proline in *Medicago sativa* and *Medicago truncatula* leaf and root tissue. Furthermore, our own data show that the proline content increases in both *Medicago truncatula* accessions TN1.11 (tolerant) and JA17 (sensitive), with a higher increase in proline content for TN1.11 than for JA17 (Phillips et al. unpublished).

Malondialdehyde accumulation is another drought stress response that has been reported as a suitable indicator for oxidative damage to membrane lipids [51]. Furthermore, the content of MDA reflects the degree of cell membrane damage and evaluates the drought tolerance abilities of plants [52].

In our study, water deficit stress caused a significant increase in the MDA content in the tested lines of *M. truncatula*, which clearly means that the plants were suffering from stress.

Additionally, the MDA accumulation was significantly lower in tolerant line TN6.18 than in the other lines under water deficit stress conditions, which suggests that it was the most protected against the oxidative stress.

Our finding is in agreement with Rajasthan et al. [53] and Premachandra et al. [46], who reported that a higher cell membrane stability index and a lower membrane damage were found in drought-tolerant lines of wheat and maize, respectively, when subjected to water stress.

In addition, Türkan et al. [54] found that the MDA content was lower in the leaves of drought-tolerant *Phaseolus acutifolius* Gray than that in drought-sensitive *P. vulgaris* L. Sairam and Srivastava [55] reported that the drought-tolerant genotypes of wheat showed lower a lipid peroxidation level.

### 3.3. Expression Analysis of DREB1B under Osmotic Stresses

Water deficit stress induces the expression of drought-responsive genes, such as the dehydration-responsive element-binding proteins (DREB) which encode transcription factors.

The *DREB1B* gene belongs to the plant-specific AP2 (APETALA2)/ERF (ethylene-responsive element-binding factor) family, is specifically induced by drought stress, plays an important role in plant growth and development [56], and is also involved in the transcriptional induction of the responsive genes under osmotic and cold stresses [57]. Furthermore, previous studies on the functional characterization of *DREB1B* in crop plants, including *Arabidopsis* [58], rice [59], *Glycine max* [60], and wheat [61] indicated that they play a crucial role in the tolerance to osmotic stress. The gene structure of *DREB1B* showed a lack of introns and a short gene length. Previous reports showed that reduced introns in genes provide the fastest process for the cell to respond to multiple abiotic stresses [62] and reduces the cost of transcription [63]. In the present study, the *DREB1B* was highly expressed in the water deficit stress-tolerant lines TN6.18 and JA17 in the roots, suggesting their role in osmotic stress, root morphogenesis, and plant development processes, as reported by Janiak et al. [64]. Our findings are in agreement with earlier studies on the *DREB* transcription factor family and their role in regulating osmotic stress responses [65,66,67], which provide evidence that these gene members play a crucial role in increasing their abiotic stress tolerance in various tissues.

## 4. Materials and Methods

### 4.1. Plant Material and Experimental Conditions

Four lines of *M. truncatula*, including two Tunisian lines TN1.11 and TN6.18, the reference line Jemalong A17 (JA17) from the Australian collection, and one Moroccan line A10 were used. The seeds collected from the pods of the selected lines were scarified using sandpaper Q60 to lift the integumentary inhibition, incubated in darkness at 4 °C for 72 h, and then transferred at 21 °C during 24 h for germination.

The germinated seeds were transplanted into black two-liter pots (Height 13.2 cm, Diameter 16.7 cm) of a sand and compost mixture (3:1. *v*/*v*) kept in a controlled growth chamber at the Center of Biotechnology of Borj Cedria, Tunisia, at a temperature of 24 °C (day) and 18 °C (night), a relative humidity of 60–80%, and a photoperiod of 16/8 h (day/night).

The plants were watered every 2 days with a Fahräeus nutrient solution [68] until the sixth leaf stage, and then they were subjected to osmotic treatments (control: 100% field capacity (FC), 30% FC, and 100 mM NaCl). Six replicates per line and per treatment were used. In order to control the water content in the pots, weighing was carried out for each pot every two days, after which the plants were watered to maintain levels of 100% and 30% FC. Furthermore, osmotic stress (induced by 100 mM NaCl) was applied to plants as described by Hdira et al. [32].

### 4.2. Growth Parameter Measurements and Physiological Assays

Several morpho-physiological parameters related to aerial parts and roots were measured for these four lines of *M. truncatula* at the flowering stage. They included the number of axes, length of stems, number of leaves, shoot fresh weight, shoot dry weight, length of roots, root fresh weight, root dry weight, ratio of root dry weight and aerial dry weight (RDW/ADW), and relative water content (RWC).

For the dry biomass measurement, the plant material was dried in an oven at 65 °C for 48 h.

The relative water content (RWC) was estimated as follows:

RWC (%) = [(LFW − LDW)/(LTW − LDW)] × 100(1)

where LFW, LDW, and LTW are the fresh, dry, and turgid weight of leaves, respectively.

For each line, four leaves were excised from plants under the three treatments, immediately weighed (LFW), and then floated overnight in water to gain turgidity. Turgid leaves were then weighed (LTW) and dried at 80 °C for 48 h.

### 4.3. Biochemical Analyses

Malondialdehyde (MDA), soluble sugars, and protein contents were determined, in the leaves and roots of the tested lines under the three treatments, as described by Hdira et al. [32]. Three replicates per line and per treatment were used. Lipid peroxidation was quantified as MDA content and was determined according to the method of Verma and Dubey [69] by measuring the absorbance of supernatant at 532 nm, and the value for non-specific absorption at 600 nm was subtracted.

The soluble sugar content was estimated based on the reliable colorimetric method described by Yemm and Willis [70] using a solution of ethanol with a concentration of 80% with anthrone–sulfuric acid. The concentration of total proteins was estimated based on the Bradford protocol [71] using bovine serum albumin as a standard.

### 4.4. Database Search and Identification of DREB1B in M. truncatula

To better understand the specific response of *M. truncatula* lines, under osmotic stress conditions, we selected one of the members of the *MtDREB* family, namely *DREB1B*. The expression of this gene was analyzed using qRT-PCR in four contrasting lines, namely TN6.18 and JA17 (tolerant lines), TN1.11 (a moderately tolerant line), and A10 (sensitive line) in different plant parts (leaves, stems, and roots). The *DREB1B* data in *M. truncatula* were obtained from the *Medicago* Hapmap PHYTOZOMEv13 database (https://phytozomenext.jgi.doe.gov/info/Mtruncatula_Mt4_0v1, accessed on February 2016) and NCBI Blast+2.2.28 (http://blast.jcvi.org/Medicago-Blast/index.cgi, accessed on February 2016) by using search queries for the “*DREB1B*” and “*Medicago truncatula*” keywords. The results of the search were used on blast against the *M. truncatula* genome (*M. truncatula* Genome Project v4.0; http://www.jcvi.org/medicago/, accessed on February 2016) [72] with the parameter values ≤ 1E^−3^ and more than 80% of coverage. We used the Pfam database (http://pfam.xfam.org/, accessed on February 2016) [73] to confirm the reliability of the candidate gene *DREB1B* based on the presence of a conserved AP2 domain.

### 4.5. RNA Extraction, cDNA Synthesis, and RT-qPCR Analysis

The total RNA extraction was from different tissues of the studied lines, as described by Zeng and Yang [74]. The evaluation of the total extracted RNA and cDNA synthesis was performed as described by Hdira et al. [32]. The quantification of the level of *DREB1B* transcripts in the different tissues of plants under control treatment and stress conditions was carried out using quantitative real time and was detected with SYBR Green (Roche, Mannheim, Germany) as described by Hdira et al. [32]. Three replicates per line and per treatment were used in all analyses.

### 4.6. Statistical Analyses

The obtained data were analyzed using a three-way ANOVA, with repeated measures using *SPSS* statistical software (version 17.0 SPSS Inc., Chicago, IL, USA). Only traits showing a significant interaction of line × treatment were retained for the remaining statistical analyses.

A comparison of the means of the analyzed parameters was performed using the Duncan’s Multiple Range Test at 5% level of probability.

The broad-sense heritability (*H*^2^) of measured parameters was estimated as the ratio of genetic variance to the sum of genetic (Vg) and environmental (Ve) variances [75]:

*H*^2^ = Vg/Vg + Ve (2)

The phenotypic correlations between the measured traits for *M. truncatula* lines under control and drought stress were estimated by calculating the Pearson correlation coefficient (r) using Proc Correlate in the SPSS software. The significance level was set at 0.05 and adjusted for multiple comparisons by Bonferroni correction [76].

The drought susceptibility index (DSI) of each trait was calculated to identify genotypes differing in their response to drought.

To analyze the responses of the studied lines under the two treatments (control and drought stress), the DSI values were subjected to a hierarchical cluster analysis (HCA) using XLSTAT software (Version 2014.5.03. Addinsoft, Paris, France).

## 5. Conclusions

Our results showed that water deficit stress influenced the morphology, physiology, and metabolic features of the four contrasting *M. truncatula* lines TN6.18, TN1.11, A10, and JA17. However, the tolerant lines TN6.18 and JA17 displayed a higher performance in terms of root biomass production as protection from damage, while the moderately tolerant line TN1.11 was the least affected in shoot biomass productivity compared to the sensitive line A10. Additionally, the results suggest that the main reason for the better drought tolerance of TN6.18 is its higher capacity for the synthesis and accumulation of osmo-protectants and its effective defense against reactive oxygen species (ROS) under stress water conditions. The ROS protection is most likely associated with the enhanced enzymatic antioxidant activity. Furthermore, osmotic stress induced *DREB1B* gene expression in different tissues, especially in roots of the tolerant lines, indicating that this gene is a positive regulator of multiple stresses in *M. truncatula*. The analyzed morpho-physiological and molecular features could be a target for breeding programs in *M. truncatula* to enhance crop yield under water deficit stress.

## Figures and Tables

**Table 1:** Effects of lines, water deficit treatments, and the interaction of line × treatment on measured traits for the studied lines of *M. truncatula* under control condition and 30% of field capacity (FC).

	Treatment		Line		Line × Treatment	
	F	*p*	F	*p*	F	*p*
Number of axes	14.29	0.00	4.86	0.01	0.01	0.01
Length of stems (cm)	165.67	0.00	65.94	0.00	0.00	0.00
Number of leaves	1553.67	0.00	277.73	0.00	0.00	0.00
Aerial fresh weight (g)	8.77	0.01	399.78	0.04	0.04	0.04
Aerial dry weight (g)	1.04	0.32	122.78	0.56	0.56	0.56
Length of roots (cm)	125.66	0.00	5.34	0.01	0.01	0.01
Root fresh weight (g)	34.77	0.00	74.80	0.00	0.00	0.00
Root dry weight (g)	0.16	0.70	48.98	0.00	0.00	0.00
Root dry weight and aerial dry weight ratio	4.05	0.06	5.16	0.03	0.03	0.03
Root water content	12.43	0.00	0.68	0.37	0.37	0.37
Chlorophyll a	2.75	0.12	1.06	0.90	0.90	0.90
Chlorophyll b	0.75	0.40	0.05	0.44	0.44	0.44
Relative growth rate (g)	18.69	0.00	3.59	0.75	0.75	0.75

F: Coefficient of Snedecor–Fisher, significant (*p* ≤ 0.05).

**Table 2:** Means of measured characters for the four lines of *M. truncatula* under control treatment and 30% of field capacity.

Number of Axes	Length of Stems	Number of Leaves	Aerial Fresh Weight	Aerial Dry Weight	Length of Roots	Root Fresh Weight	Root Dry Weight	Root Dry Weight and Aerial Dry Weight Ratio
Trait/line (100%FC)								
1 ± 0 ^c^	54 ± 1.15 ^a^	38 ± 3.51 ^c^	1.23 ± 0.27 ^fg^	0.16 ± 0.02 ^f^	43.67 ± 2.96 ^bc^	0.47 ± 0.06 ^f^	0.13 ± 0.01 ^fg^	0.81 ± 0.11 ^b^
4.67 ± 0.88 ^a^	35.33 ± 0.88 ^c^	79.67 ± 0.88 ^b^	4.26 ± 0.19 ^c^	0.99± 0.07 ^cd^	49 ± 4.04 ^ab^	4.52 ± 0.36 ^b^	0.73 ± 0.1 ^abc^	0.73 ± 0.16 ^bc^
4 ± 1.53 ^ab^	51 ± 0.58 ^ab^	151.67 ± 1.45 ^a^	10.33 ± 0.36 ^a^	2.95 ± 0.34 ^ab^	48 ± 1.73 ^a^	6.37 ± 0.46 ^a^	0.78 ± 0.08 ^ab^	0.26 ± 0.01 ^ef^
1 ± 0 ^c^	16 ± 1.15 ^fg^	35.33 ± 2.60 ^cd^	1.02 ± 0.13 ^f^	0.11 ± 0.02 ^g^	27 ± 4.04 ^d^	0.19 ± 0.03 ^gh^	0.01 ± 0 ^h^	0.09 ± 0.02 ^h^
Trait/line (30%FC)								
1 ± 0 ^c^	28 ± 5.29 ^cde^	26.33 ±2.4 ^e^	0.72 ± 0.39 ^efgh^	0.03 ± 0.01 ^h^	19 ± 5.20 ^defgh^	0.30 ± 0.04 ^fg^	0.15 ± 0 ^f^	0.66 ± 0.03 ^a^
1 ± 0 ^c^	21.90 ± 1.10 ^d^	17 ± 0 ^f^	2.63 ± 0.12 ^d^	1.14 ± 0.03 ^c^	27 ± 1.76 ^ef^	1.85 ± 0.29 ^cd^	0.37 ± 0.08 ^d^	0.32 ± 0.07 ^defg^
1 ± 0 ^c^	19.50 ± 1.61 ^def^	11 ± 0.58 ^h^	9.66 ± 0.54 ^ab^	3.05 ± 0.31 ^a^	15.67 ± 1.20 ^e^	2.79 ± 0.67 ^bc^	0.80 ± 0.06 ^a^	0.26 ± 0 ^e^
1 ± 0 ^c^	9.33 ± 0.88 ^h^	19 ± 2.52 ^efg^	1.33 ± 0.03 ^e^	0.47 ± 0.47 ^e^	27 ± 2.03 ^efg^	1.06 ± 0.14 ^e^	0.27 ± 0.04 ^de^	0.57 ± 0.16 ^bcd^

Standard errors of the mean SEM were evaluated. The means of each trait followed by the same or common letters are not significantly different among the studied lines according to Duncan’s multiple range test at 5%.

**Table 3:** Genetic (Vg) and environmental (Ve) variances and heritability (*H*^2^) of measured traits for *M. truncatula* lines under control treatment and water deficit stress (30% FC).

Treatment/Trait	Control			30% FC		
	Vg	Ve	*H* ^2^	Vg	Ve	*H* ^2^
Number of axes	3.00	2.33	0.56	0.00	0.00	0.00
Length of stems	302.86	2.83	0.99	52.27	24.43	0.68
Number of leaves	2940.02	16.50	0.99	36.89	9.33	0.80
Aerial fresh weight	18.78	0.19	0.99	16.94	0.34	0.98
Aerial dry weight	1.73	0.09	0.95	1.74	0.08	0.95
Length of roots	93.14	33.33	0.74	0.00	26.75	0.00
Root fresh weight	9.21	0.26	0.97	1.01	0.40	0.72
Root dry weight	0.15	0.02	0.90	0.08	0.01	0.90
Root dry weight and aerial dry weight ratio	0.13	0.03	0.82	9.89	8.45	0.54

**Table 4:** Matrices of correlations between measured traits for the studied lines of *M. truncatula* under control treatment (down diagonal) and 30% of field capacity (up diagonal).

	NA	LS	NL	AFW	ADW	LR	RFW	RDW	Ratio
NA	1.00	−0.17	0.12	−0.12	−0.08	−0.08	−0.08	−0.17	0.14
LS	0.17	1.00	0.41	0.01	−0.05	0.28	−0.15	−0.10	0.61 *
NL	0.61 *	0.44	1.00	−0.76 **	−0.83 **	0.42	−0.74 **	−0.81 **	0.63 *
AFW	0.59 *	0.44	0.99 **	1.00	0.98 **	−0.12	0.76 **	0.95 **	−0.39
ADW	0.58 *	0.44	0.98 **	0.99 **	1.00	−0.15	0.80 **	0.96 **	−0.48
LR	0.67 *	0.67 *	0.54	0.53	0.50	1.00	−0.18	−0.14	−0.12
RFW	0.79 **	0.38	0.94 **	0.93 **	0.91 **	0.66 *	1.00	0.83 **	−0.52
RDW	0.73 **	0.38	0.82 **	0.80 **	0.79 **	0.67 *	0.95 **	1.00	−0.46
Ratio	0.03	0.52	−0.21	−0.23	−0.26	0.51	−0.04	0.20	1.00

* Significant correlation at 0.05 level and ** Significant correlation at the 0.01 level.

**Table 5:** Effects of line, treatment, and the interaction line × treatment on biochemical parameters for studied lines of *M. truncatula* under control treatment and drought stress.

	MDA		Proteins		Soluble Sugar	
	F	*p*	F	*p*	F	*p*
Line	1.28	0.31	7.15	0.00	3.39	3.39
Treatment	19.75	0.00	0.78	0.39	24.00	0.00
Line × treatment	2.50	0.10	15.99	0.00	16.34	0.00

Coefficient of Snedecor–Fisher with significance at *p* ≤ 0.05 (F-value).

**Table 6:** Effects of line, tissue, treatment, the interaction of line × tissue, the interaction line × treatment (treat), the interaction of tissue × treatment, and the interaction of line × tissue × treatment on the expression of the *DREB1B* gene for the studied lines of *M. truncatula* under control treatment, 30% FC, and 100 mM NaCl.

	F	*p*
Tissue	324.70	0.00
Line	359.43	0.00
Treatment	143.53	0.00
Tissue × line	325.19	0.00
Tissue × Treatment	103.07	0.00
Line × Treatment	131.89	0.00
Tissue × line × Treatment	107.59	0.00

F is the coefficient of Snedecor–Fisher with significance at *p* ≤ 0.05.

## Data Availability

Data is contained within the article and supplementary material.

## Supplementary Materials

The following are available online at https://www.mdpi.com/article/10.3390/plants10102114/s1, Figure S1: Variability of expression analyses of *DREB1B* in leaves, stems, and roots of the studied lines of *M. truncatula* under control treatment and 100 mM NaCl, Table S1: Minimum, maximum, and means of measured characters for the four lines of *M. truncatula* under control treatment and 30% of field capacity, Table S2: Primer sequences information.
