# Supplementary material for: Evaluation of the Morpho-Physiological, Biochemical and Molecular Responses of Contrasting Medicago truncatula Lines under Water Deficit Stress
Source: Plants (Basel). 2021 Oct 6;10(10):2114. doi: 10.3390/plants10102114 (PMC8537959; doi:10.3390/plants10102114)
Supplement: Supplementary file 1 [file plants-10-02114-s001.zip › plants-1320043-supplementary.pdf]

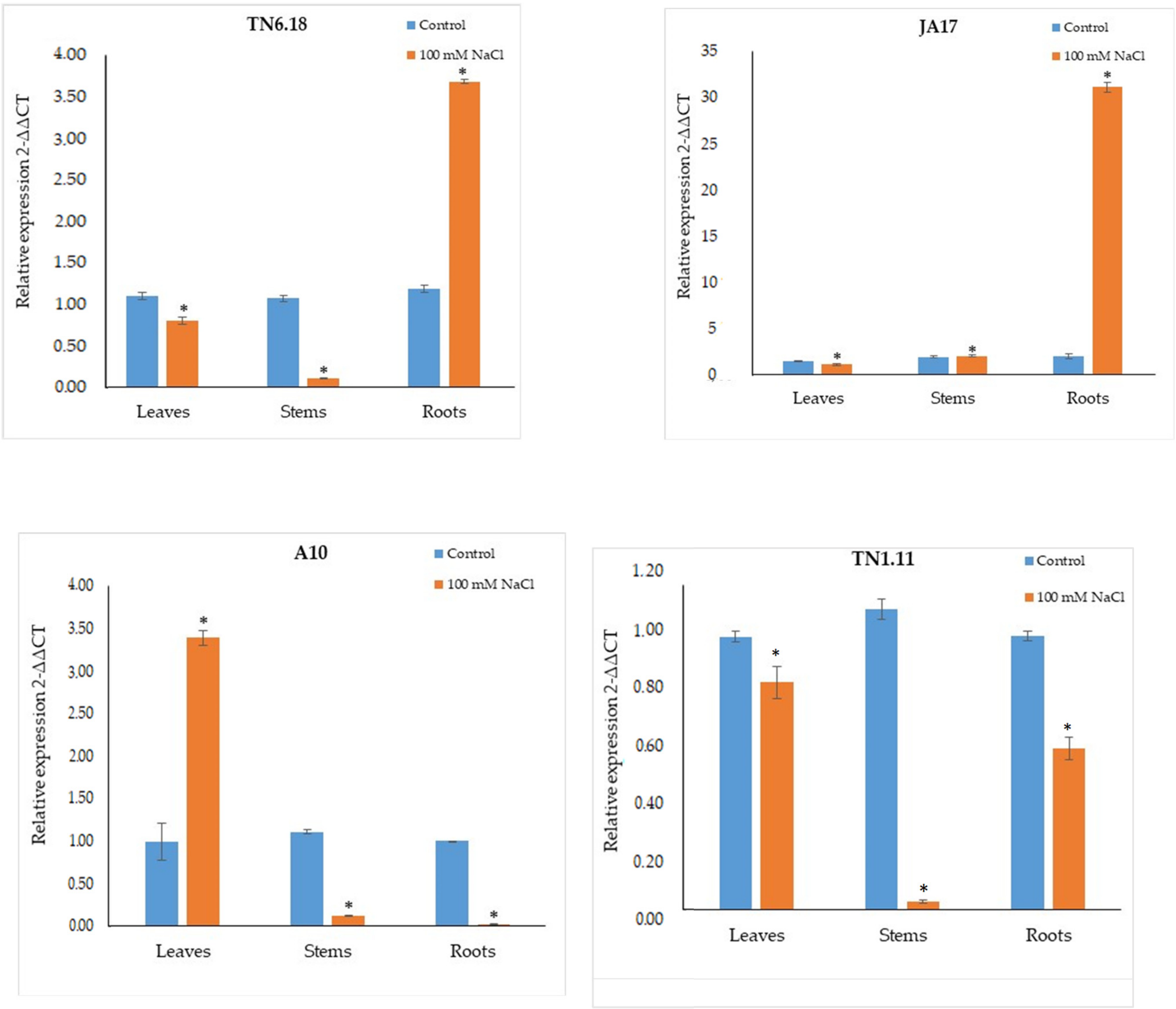

Figure S1. Variability of expression analyses of DREB1B in leaves, stems and roots of the studied lines of *M. truncatula* under control treatment and 100 mM NaCl.

Table S1. Minimum, maximum, means and standard error of measured characters for the four lines of *M. truncatula* under control treatment and 30% of field capacity.

		Minimum	Maximum	Mean	Standard error	F	*P*
Number of axes	Control	0.00	6.00	2.67	2.19	4.86	0.03
	Drought	1.00	1.00	1.00	0.00	-	-
Length of stems	Control	14.00	56.00	39.08	15.83	321.68	0.00
	Drought	8.00	36.00	19.68	8.20	7.42	0.01
Number of leaves	Control	31.00	154.00	76.17	49.21	535.55	0.00
	Drought	10.00	31.00	18.33	6.29	12.86	0.00
Aerial fresh weight	Control	0.79	10.96	4.21	3.94	292.57	0.00
	Drought	0.18	10.72	3.59	3.77	150.13	0.00
Aerial dry weight	Control	0.08	3.60	1.05	1.23	59.12	0.00
	Drought	0.01	3.65	1.17	1.23	64.60	0.00
Length of roots	Control	20.00	54.00	41.92	10.47	9.38	0.01
	Drought	10.00	28.00	16.83	4.61	0.25	0.86
Root fresh weight	Control	0.14	7.24	2.89	2.79	106.01	0.00
	Drought	0.21	4.13	1.50	1.11	8.56	0.01
Root dry weight	Control	0.01	0.93	0.41	0.38	28.67	0.00
	Drought	0.14	0.90	0.40	0.27	28.28	0.00
Root dry weight and aerial dry weight ratio	Control	0.06	1.07	0.49	0.37	14.87	0.00
	Drought	0.19	13.88	2.19	4.07	4.52	0.04
Root water content	Control	47.70	81.62	73.14	10.53	1.12	0.40
	Drought	33.73	81.87	55.00	14.11	0.80	0.53
Chlorophyll a	Control	9.80	22.93	19.27	3.38	0.54	0.67
	Drought	11.27	20.78	17.11	2.61	0.77	0.54
Chlorophyll b	Control	6.51	19.20	11.26	4.05	0.55	0.66
	Drought	5.99	17.39	9.84	3.43	0.45	0.73
Relative growth rate (g)	Control	0.04	0.10	0.07	0.02	3.60	0.07
	Drought	0.02	0.07	0.04	0.02	0.90	0.48

Coefficient of Snedecor-Fisher with significance at $P \leq 0.05$ (F-value).

Table S2. Primer sequences information.

Name of gene	Left primer (5'..............3')	Right primer (5'..............3')
DREB 1B	TGGTTCTGAGTCTGCGGATT	TCAACCCTTCTGCCATGCTA
**Control genes**		
β-TUB	TTTGCTCCTCTTACATCCCGTG	GCAGCACACATCATGTTTTTGG
ACT2	TCAATGTGCCTGCCATGTATGT	ACTCACACCGTCACCAGAATCC
